# Anthropogenic aerosol drives uncertainty in future climate mitigation efforts

**DOI:** 10.1038/s41598-019-52901-3

**Published:** 2019-11-12

**Authors:** E. J. L. Larson, R. W. Portmann

**Affiliations:** 1 Earth System Research Laboratory, National Oceanic and Atmospheric Administration, Boulder, Colorado USA; 2 Present Address: Department of Organismic and Evolutionary Biology, Harvard University, Cambridge, MA 02138 USA

**Keywords:** Projection and prediction, Climate-change mitigation

## Abstract

The 2016 Paris agreement set a global mean surface temperature (GMST) goal of not more than 2 degrees Celsius above preindustrial. This is an ambitious goal that will require substantial decreases in emission rates of long-lived greenhouse gasses (GHG). This work provides a mathematical framework, based on current state of the art climate models, to calculate the GHG emissions consistent with prescribed GMST pathways that meet the Paris agreement goal. The unique capability of this framework, to start from a GMST timeseries and efficiently calculate the emissions required to meet that temperature pathway, makes it a powerful resource for policymakers. Our results indicate that aerosol emissions play a large role in determining the near-term allowable greenhouse gas emissions that will limit future warming to 2 °C, however in the long term, drastic GHG emissions reductions are required under any reasonable aerosol scenario. With large future aerosol emissions, similar to present day amounts, GHG emissions need to be reduced 8% by 2040 and 74% by 2100 to limit warming to 2 °C. Under a more likely low aerosol scenario, GHG emissions need to be reduced 36% and 80% by 2040 and 2100, respectively. The Paris agreement Intended Nationally Determined Contributions are insufficient to meet this goal.

## Introduction

A pressing question in this era of global change is how much GHG emissions need to be reduced to limit global warming. Although no human-caused climate perturbation is desirable, the 2015 Paris agreement set a GMST goal of below 2 °C compared to preindustrial levels, with an effort to limit warming to 1.5 °C. While some authors suggest that the 1.5 degree goal is still attainable^1,2^ many others argue that it is unlikely without immediate and drastic reductions in emissions or active carbon sequestration, i.e. negative emissions^3,4^. Current policy efforts in the form of INDCs are not predicted to achieve a 1.5 or even 2 °C GMST anomaly by end of century^5–7^. There are arguments that overshooting scenarios, i.e. situations in which the GMST rises above the goal temperature for some period before returning to the goal, can help achieve temperature goals by releasing energy from the Earth system to space via long wave radiation. Overshooting scenarios can have converging climate properties similar to non-over-shooting scenarios with the same cumulative CO_2_ emissions depending on the time horizon^8^. However, there is a growing consensus that overshooting scenarios for end of century temperature goals will require negative emissions^4,5,9^. Negative emissions scenarios are not reliable plans for global warming mitigation. Not only are they susceptible to future political will, the technology is also currently not developed or tested^10^. Furthermore, solar geoengineering is not a reliable offset for GHG radiative forcing. There are many potential risks to humanity associated with solar geoengineering, including; acid rain, changes to the hydrological cycle, and termination shock, i.e. a dramatic warming if solar engineering is stopped^10^. As such, the safest plan for limiting future warming is to limit GHG emissions.

The year that the Paris agreement was signed, 2016, saw record global warming of 1.23 °C above preindustrial, and the last decade, 2009–2018, has warmed by 0.3 °C alone^11^. Although some of this recent warming was likely an accelerated response to the 2000’s global warming slowdown, the current warming rate need only be maintained for a decade before we reach 1.5 °C. Thus, in this paper, we focus on the emission limits that would lead to the more attainable goal of a 2 °C above preindustrial.

## Results

**Figure 1:**
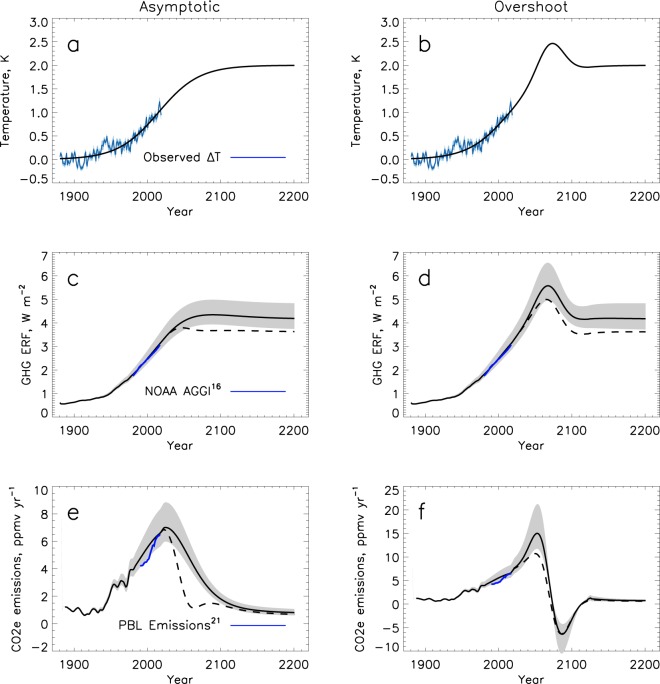
(**a**,**b**) The historical GMST anomaly^24–27^ (blue) along with assumed future asymptotic and overshoot temperature pathways (black). (**c**,**d**) The allowed GHG effective radiative forcing (ERF) calculated from the fitted temperature time series using the CMIP5 kernels (black) and two different aerosol scenarios. Dashed lines indicate the reduced anthropogenic aerosol scenario. Grey shading indicates the range of ERF calculated from the inter 2/3 s of CMIP5 model kernels. The NOAA GHG forcing^16^ during the past few decades is shown in blue. (**e**,**f**) The past and future equivalent CO_2_ emissions (CO_2_e) corresponding the GHG ERF time series. Recent CO_2_e emission estimates from the Netherlands Environmental Assessment Agency (PBL)^19^ are plotted in blue for comparison. For reference, 1ppmv equals about 7.8 Pg of CO_2_.

While most climate studies^5,12,13^ start with a GHG scenario and calculate the GMST response, the technique presented here does the opposite, and starts with a policy relevant temperature goal, 2 °C above preindustrial, and calculates the GHG emissions that would create that temperature. We consider two temperature pathways that approach 2 °C by 2100 and maintain 2 °C out to 2200; a logistic function that exponentially asymptotes, and an overshooting pathway (Fig. 1a,b). We extend our analysis to 2200 to ensure that the temperatures are not simply passing through the end of century goal, but are maintained. Using a novel kernel method^14^, based on the 5^th^ Climate Model Intercomparison Project (CMIP5)^15^ GMST response to CO_2_ forcing, we calculate the total effective radiative forcing (ERF) that the average CMIP5 climate model would need to follow to achieve these GMST pathways. The allowed GHG ERF is calculated by subtracting an assumed aerosol scenario (Fig. 1c,d) from the total ERF. The NOAA Adjusted Greenhouse Gas Index (AGGI)^16^ over the last 40 years is used to calibrate the historical aerosol ERF.

**Figure 2:**
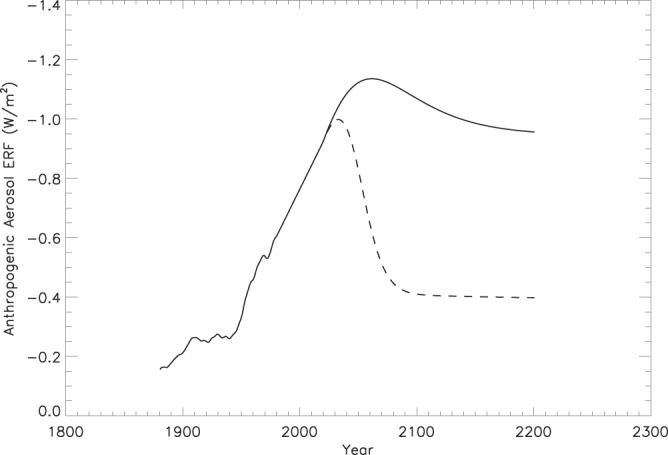
The anthropogenic aerosol forcing scenarios assumed in Figs 1 and 3. The solid line is the high aerosol scenario and the dashed line is the low aerosol scenario.

Aerosols, which scatter light back to space, negatively force the climate system and offset some of the GHG ERF. Larger future aerosol emissions would allow more GHG emissions for the same temperature change. We partition the future ERF pathway into GHG, volcanic, and anthropogenic aerosol. While future volcanic eruptions are impossible to predict, not including a volcanic term would bias the results. Thus, we assume a constant volcanic aerosol ERF equal to the global mean volcanic aerosol forcing over the historical period from 1850–2011 of −0.35 (W/m^2^)^17^. Adding variability to future volcanic forcing does not change the allowed cumulative GHG emissions. The amount of future anthropogenic aerosol assumed greatly affects the GHG emissions allowable under a 2 °C temperature pathway. We consider two future scenarios, a high aerosol scenario in which the anthropogenic aerosols offset a similar fraction of the GHG forcing as they do in 2017 and a low aerosol scenario in which the aerosol forcing is reduced to 0.4 W/m^2^ over the next 60 years. The latter is chosen to be consistent with the CMIP5 RCP45 projections. The aerosol radiative forcing at the end of the century in these two scenarios are 1.17 and 0.4 W/m^2^, respectively (Fig. 2). The low aerosol scenario is considered a more realistic scenario, as emissions of anthropogenic aerosol are expected to decrease with decreased GHG emissions and increased pollution controls. It is unlikely that GHG emissions will decrease without related reductions in aerosol emissions.

Using the analytical formulae to convert GHG ERF to CO_2_ concentration, and the atmospheric lifetime of CO_2_, we calculate the corresponding equivalent CO_2_ (CO_2_e) emissions that would result in the calculated GHG forcing (Fig. 1e,f) (see methods). Here we use a quad exponential based on empirical fits to a carbon cycle multi-model intercomparison to calculate the lifetime of CO_2_^18^. The allowed GHG emissions calculated in this study are sensitive to the CO_2_ lifetime, and an increase in the CO_2_ lifetime would decrease the allowed emissions calculated using this method. However, our independent estimate of historical GHG emissions derived from the temperature and CMIP5 models compares well with a bottom up CO_2_e emissions estimate^19^ (Fig. 1e,f). GHGs have different lifetimes and thus will affect climate on different time scales. Here we use CO_2_e to represent GHG emissions, knowing that CO_2_e does not capture the complex set of scenarios that can evolve with different ratios of GHG emissions. However, CO_2_e is the best metric available for current predictions starting from temperature and forcing requirements and provides a robust estimate of GHG emissions that is comparable with past literature. Under the asymptotic temperature pathway and high aerosol burden, the global CO_2_e emissions peak at 7.0 ppmv yr^−1^ or 55 Gt yr^−1^ in 2024. Under reduced aerosols, CO_2_e emissions peak at 6.8 ppmv yr^−1^ or 53 Gt yr^−1^ in 2021. The overshoot temperature pathway allows for substantial increase in CO_2_e emissions for decades, however it also requires prolonged and unlikely negative emissions late this century.

Under either aerosol scenario, GHG emissions need to reduce drastically by the end of the century to limit warming to 2 °C (Fig. 1e,f). By 2100, the allowed CO_2_e emissions in the high and low aerosol scenarios are 11 and 14 Pg/yr, respectively. The assumed aerosol scenario mostly affects the near-term emissions limits, with high aerosol emissions simply buying time before GHG reductions are necessary.

**Figure 3:**
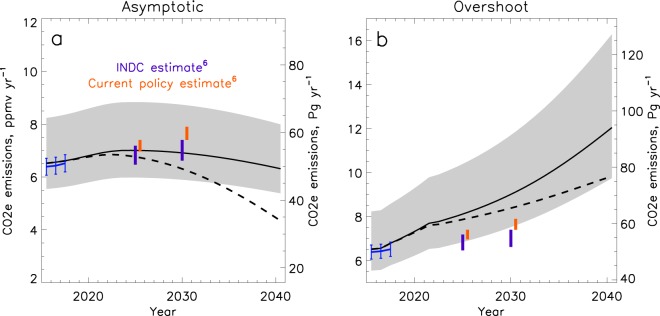
Near-term allowed CO_2_e emissions limits for high (solid line) and low (dashed line) aerosol scenarios for each temperature pathway along with estimates of recent bottom up emissions^19^ (blue) and future emissions from INDCs (purple) and current policies (orange) in 2025 and 2030^6^. Grey shading indicates the range of ERF calculated from the inter 2/3 s of CMIP5 model kernels.

Figure 3 highlights the near-term future of the CO_2_e emission pathways shown on Fig. 1. The uncertainty estimates indicate the emissions required by the inter 2/3 CMIP5 models to recreate the prescribed temperature pathways. This spread reflects the range of model climate sensitivities. However, all of the models respond to increased GHG emissions with increased warming. Although this range encompasses both the current policy and INDC estimates, the uncertainty presented in Fig. 3 should not be interpreted as allowable emissions limits before mitigation. The GHG ERF and emissions derived from the median CMIP5 model response is consistent with observed GHG forcing and emissions estimates^16,19^. Therefore, following the median emissions pathway gives the best chance at following the prescribed temperature pathways.

To be consistent with 2 °C warming in 2100, CO_2_e emissions need to decrease by 8% in 2040 and 74% in 2100 assuming no reductions in aerosols (Fig. 2a, Table 1). The estimated INDC emissions in 2025 and 2030 are consistent with the median emission pathway. The current policy estimates and INDCs indicate increasing emissions between 2025 and 2030 however, which is inconsistent with a 2 °C temperature limit. CO_2_e emissions need to start decreasing in the near future to limit warming to 2 °C, and every year they continue to rise, that temperature goal becomes less likely. The overshoot pathway, even under the low aerosol scenario, allows for larger GHG emissions than current policy estimates. However, this scenario would require reliance on negative emissions later in the century (Fig. 2b).

**Table 1:** Percent change in CO_2_e emissions between the indicated years under future climate scenarios.

Scenario	2025–2030	2020–2040	2020–2100
Current policy estimates^6^	+6.5	NA	NA
INDC^6^	+2.8	NA	NA
High aerosol emissions	−1.5	−7.8	−74
Low aerosol emissions	−7.3	−36	−80

If anthropogenic aerosol pollution is reduce as expected, the emissions reductions necessary to reach the 2 °C temperature goal are much greater. Under our clean air (low aerosol) scenario, which has 65% less anthropogenic aerosol forcing in 2100, CO_2_e emissions need to be reduced by 40% by 2040 and 82% by 2100 (Table 1). These are substantial reductions in emissions and well outside of the near term INDC estimates, however these may still be feasible. A recent study finds that phasing out the current fossil fuel infrastructure over the next 40 years might limit warming to 1.5 C; however, waiting until 2030 to start mitigation greatly reduces that likelihood^2^.

**Figure 4:**
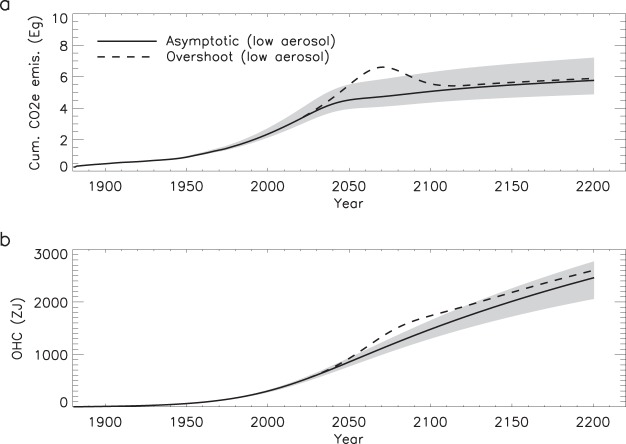
Cumulative CO_2_e (**a**) and ocean heat content (OHC) (**b**) associated with each temperature pathway (see Fig. 1). Grey shading indicates the range of OHC from the asymptotic temperature pathway and the inter 2/3 of CMIP5 kernels.

It is well known that the GMST is proportional to the cumulative CO_2_e emissions^20,21^, so it is not surprising that the cumulative CO_2_e emission for the two temperature pathways converge after 2100 (Fig. 4a). Between 2019 and 2100, allowed cumulative CO_2_e emissions to achieve 2 °C warming under our high aerosol scenario are 2900 (2470–3650) Pg. If the anthropogenic aerosol burden is reduced by about 65%, the cumulative allowed CO_2_e emissions are 1820 (1470–2440) Pg by 2100.

Global surface warming is not the only climate risk associated with GHG emissions. Along with surface warming comes precipitation changes, ocean acidification, and global sea level rise^4^. We find that the change in ocean heat content (OHC) in the overshoot temperature pathway is about 300 ZJ larger in 2100 than in the asymptotic pathway (Fig. 4b). This translates into 3 cm difference in thermosteric seal level in 2100 between the two scenarios. Consistent with our findings, the GFDL model indicated strong pathway-dependence of sea level rise in northern North American cities, with overshoot forcing producing up to 10 cm of additional thermosteric sea level rise by 2100 relative to stabilization forcing^12^. Furthermore, we note that although the GMST remains constant at 2 °C under these temperature pathways, the OHC and sea level show no signs of stabilization by 2200. While temperature goals are useful, it is important to be mindful of the many additional climate risks associated with elevated CO_2_ and other GHG concentrations.

## Discussion

Future aerosol emissions play a crucial role in determining the allowable GHG emissions. However, the heterogeneity in space and time of future aerosol emissions adds a great deal of uncertainty to estimating the radiative forcing. Variability in the timing of volcanic eruptions will create variability in the ERF and climate signal from aerosols but will not affect the cumulative allowed GHG emissions to reach the 2 °C temperature target. Regional changes in the type and amount of anthropogenic aerosol emission could change the anthropogenic aerosol ERF directly through the amount of aerosol emitted and indirectly through changes in efficacy which is tied to the location and type of emitted aerosol. Recent observations suggest differing regional trends in aerosol optical depth and radiative forcing^22,23^. For these reasons, we consider a large range of future aerosol ERF in calculating our allowed GHG emissions.

Global GHG emissions and atmospheric concentrations are still on the rise^16^. Each year that we persist with the “business as usual” plan puts the Paris agreement temperature goals further out of reach. Modest GHG emission reductions, beyond the INDCs, in the near future could keep us on track to limiting warming to 2 °C, however aerosol emissions add a great deal of uncertainty about the timing and rate of GHG reductions. Cleaning up anthropogenic aerosol emissions, which is a likely scenario under GHG emissions mitigation, will require much larger reductions in GHG emissions in the near-term and slightly larger reductions in the long term to limit global warming to 2 °C above preindustrial. Regardless of the aerosol scenario, GHG emissions need to drastically reduce by 2100, with high aerosol emissions only buying a little time.

## Methods

### Calculating GHG effective radiative forcing from GMST

We fit Earth’s past GMST and the future pathway to 2 °C above preindustrial with a logistic function. Logistics make a very good approximation for past and current warming while constraining future warming to a goal temperature. We also include an overshoot scenario modeled as the logistic function, for consistency in past and current warming, with an added Gaussian function peaking in 2070 at 2.45 C. The functional forms of each fitted temperature pathway are as follows:

1 $${\rm{Asymptotic}}:\,{\rm{T}}=2\ast {(1+{e}^{(-0.036\ast (year-2014.5))})}^{-1}$$

2 $${\rm{Overshoot}}:{\rm{T}}={\rm{Logistic}}+0.7\ast {{\rm{e}}}^{(-0.00167\ast {({\rm{year}}-2070)}^{2})}$$

The slope (0.036) and midpoint (2014.5) of the logistic function were determined using a least-squares fit to the mean observed temperature anomaly time series from the following sources: NOAA^24^, GISS^25^, Hadcrut^26^, and Berkeley^27^.

We use the temporal kernel method^14^ to calculate the historical ERF from observed global mean surface temperatures based on the CMIP5 median kernel from the 4xCO_2_ experiments^28^. This method uses the response of the global mean surface temperature (*T)* to a radiative forcing step change experiment from the CMIP5 models to calculate a temporal kernel (***t***). We fit a function consisting of two exponentials to the temperature response in that step change experiments to extend our kernels out to 500 years^14^. Step change experiments, such as 4xCO_2_, in the CMIP5 simulations have constant ERF (*F*_0_). Here we use the median value from the models, *F*_0_ = 7.14 W/m^2^. In Eq. 3, *i*, is the year since the start of the simulation and *j*, is the year since the change in forcing. Here, *T*_*i*_, is the total temperature response in year *i* to the scaled changes in forcing in years *j* = 0 to *i*, with *t*_*i-j*_, being the temperature response in year *i* to the change in forcing in year *j*. *ΔF*_*j*_ is the change in forcing from the previous year.

3 $${T}_{i}=\,\mathop{\sum }\limits_{j=0}^{i}{t}_{i-j}\frac{\Delta {F}_{j}}{{F}_{0}}$$

Equation 4 is simply Eq. 3 written in matrix notation, where ***T*** and ***ΔF*** are one-dimensional vectors and ***t*** is a two-dimensional square matrix.

4 $${\boldsymbol{T}}={\boldsymbol{t}}\Delta {\boldsymbol{F}}/{F}_{0}$$

Equation 4 can be inverted to create Eq. 5, which calculates the change in forcing for a given temperature time series.

5 $$\Delta {\boldsymbol{F}}={F}_{0}{{\boldsymbol{t}}}^{-1}{\boldsymbol{T}}$$

Finally, the ERF time series can be calculated as the sum of the forcing step changes.

6 $${F}_{i}=\mathop{\sum }\limits_{j=0}^{i}\Delta {F}_{j}$$

The kernel functions (***t***) derived from the CMIP5 4xCO_2_ step change experiments for global mean surface temperature and ocean heat content are available online (https://github.com/larsonej/CMIP5_kernels). Here, the historical ERF is calculated from the observed temperature time series and is primarily the net forcing from the large positive GHG forcing and large negative forcing from volcanic and anthropogenic aerosols. There are many other forcing agents, such as ozone and land use change, that are small compared to these two.

The GHG ERF is calculated by subtracting an aerosol ERF from the total ERF calculated above from the temperature anomalies. Here we use −0.35 W/m^2^ of constant volcanic aerosol forcing, which is the global mean forcing between 1850 and 2011 from the IPCC AR5^17^. Although, we cannot predict future volcanic eruptions, assuming zero future volcanic forcing would bias our results. Using the mean volcanic forcing ignores the variability associated with volcanic events, but produces an energy budget that is consistent with past volcanic activity.

Published estimates are used for the past anthropogenic aerosol from 1880–2011^29^. Between 1980 and 2018, we used a linearly decreasing anthropogenic aerosol term to −0.93 W/m^2^. This is done to both smooth variability over the past four decades, extend the time series, and to create a GHG ERF that is consistent with the NOAA AGGI^16^ (Fig. 1b). This anthropogenic aerosol estimate is consistent in magnitude and trend with other published estimates^30,31^. We considered two future anthropogenic aerosol emission scenarios. In the first scenario, the aerosol burden remains at or above present-day levels into the future and is scaled to the GHG forcing. The second scenario greatly reduces the anthropogenic aerosol burden to −0.4 W/m^2^ by the end of the century (Fig. 2), roughly consistent with the aerosol burden in the RPC4.5 scenario.

### Calculating emissions from GHG ERF

We use the logarithmic CO_2_ forcing equation to calculate the equivalent CO_2_ (CO_2_e) concentration, *C*_*i*_, from the GHG ERF time series, *F*_*i*_^32^. The logarithmic formula presented here is for the adjusted radiative forcing, however, we are applying it to the ERF. Previous work has also showed that the instantaneous forcing follows a logarithmic relationship^33^. The assumption that the CO_2_ effective and adjusted radiative forcings are equivalent creates some uncertainty in the estimate. Zhang and Huang find that the effective and adjusted radiative forcings are 6.4 and 7.3 W/m^2^, or about 15% different in the CMIP5 models they assessed^34^. This suggests that this method may be underestimating the allowed GHG emissions. As long as the adjustments scale linearly with the forcing, this can be overcome by using the adjusted F_0_ in the kernel method as opposed to the effective F_0_. The kernels in this method are using an F_0_ of 7.14 W/m^2^ based on the median CMIP5 y-intercept of 150-year regressions of ***Δ****T* and *N*, the top of atmosphere radiative imbalance. The logarithmic formula for a quadrupling of CO_2_ provides 7.4 W/m^2^ of adjusted RF, or 0.26 W/m^2^ different. Thus, the underestimate is expected to be about 3.5 percent. This is small compared to the substantial spread in the CMIP5 model calculated forcing, which is encompassed in the uncertainty estimates in the figures.

7 $${C}_{i}=278\,{e}^{(\frac{{F}_{i}}{5.35})}$$

To convert the CO_2_e concentration to allowed emissions, we need to consider the lifetime of CO_2_ in the atmosphere. The remaining fraction of CO_2_e, *X*, is modeled as a sum of exponentials with weights (*w* = 0.2173, 0.224, 0.2824, 0.2763) and lifetimes (*t* = 394.4, 36.54, 4.304 years) from a multi-model analysis^18^.

8 $${X}_{i}={w}_{0}+\mathop{\sum }\limits_{j=1}^{3}{w}_{j}\,{e}^{(\frac{-i}{{t}_{j}})}$$

The total concentration of CO_2_e in year *i* given an emission timeseries can then be written as

9 $${C}_{i}=\mathop{\sum }\limits_{j=0}^{i}{X}_{i-j}{E}_{j}$$

where *X*_*i-j*_ is the fraction of CO_2_e emitted in year *j* that remains in year *i*, and *E*_*j*_, is the emission in year *j*. Similar to the temporal kernel method, *X*, can be treated as a kernel and Eq. 9 can be written in matrix form and inverted as follows,

10 $${\boldsymbol{E}}={\boldsymbol{C}}{{\boldsymbol{X}}}^{-1}$$

The allowed CO_2_e emissions are then calculated using the inverted kernel and the concentration time series.

### OHC from ERF

Ocean heat content (OHC) consistent with each temperature pathway is calculated from the historical and future ERF using the temporal kernel method^14,28^ described above.
